# Survival times are similar among patients with peritoneal, hematogenous, and nodal recurrences after curative resections for gastric cancer

**DOI:** 10.1002/cam4.3208

**Published:** 2020-06-08

**Authors:** Koichi Sawaki, Mitsuro Kanda, Seiji Ito, Yoshinari Mochizuki, Hitoshi Teramoto, Kiyoshi Ishigure, Toshifumi Murai, Takahiro Asada, Akiharu Ishiyama, Hidenobu Matsushita, Chie Tanaka, Daisuke Kobayashi, Michitaka Fujiwara, Kenta Murotani, Yasuhiro Kodera

**Affiliations:** ^1^ Department of Gastroenterological Surgery (Surgery II) Nagoya University Graduate School of Medicine Nagoya Japan; ^2^ Department of Gastroenterological Surgery Aichi Cancer Center Nagoya Japan; ^3^ Department of Surgery Komaki Municipal Hospital Komaki Japan; ^4^ Department of Surgery Yokkaichi Municipal Hospital Yokkaichi Japan; ^5^ Department of Surgery Konan Kosei Hospital Konan Japan; ^6^ Department of Surgery Ichinomiya Municipal Hospital Ichinomiya Japan; ^7^ Department of Surgery Gifu prefectural Tajimi Hospital Tajimi Japan; ^8^ Department of Surgery Okazaki City Hospital Okazaki Japan; ^9^ Department of Surgery Tosei General Hospital Seto Japan; ^10^ Biostatistics Center Graduate School of Medicine Kurume University Kurume Japan

**Keywords:** gastric cancer, prognosis, recurrence

## Abstract

**Background:**

The three dominant recurrence patterns of gastric cancer are peritoneal, hematogenous, and nodal recurrence. Correlation between initial recurrence site and prognosis is poorly understood, particularly after standardization of postoperative S‐1 adjuvant chemotherapy.

**Methods:**

We analyzed a multi‐institutional database of 3484 patients who underwent gastrectomy for gastric cancer between 2010 and 2014. Patients who experienced recurrences after curative gastrectomy classified into peritoneal, hematogenous, or nodal recurrence groups, according to their initial recurrence sites, and their prognoses were compared.

**Results:**

We included 313 patients in the analysis, of whom 190 patients (63%) were treated with postoperative adjuvant chemotherapy. Pathological disease states were stage I: n = 20 (6%), stage II: n = 62 (20%), and stage III: n = 231 (74%). Patients were categorized into groups by peritoneal (n = 127), hematogenous (n = 123), and nodal (n = 63) recurrence. The peritoneal recurrence group tended to have longer recurrence‐free survival, but shorter post‐recurrence survival, than the other two groups. Median disease‐specific survival after curative resection by group were peritoneal: 25.8 months, hematogenous: 29.0 months, and nodal: 27.8 months (peritoneal vs hematogenous, *P* = .152; hematogenous vs nodal, *P* = .955; peritoneal vs nodal, *P* = .213).

**Conclusions:**

Prognoses after curative resection for gastric cancer were similar among patients with peritoneal, hematogenous, or nodal recurrences.

## Graphical abstract

We used a multi‐institutional dataset to see whether prognosis and site of initial recurrence after curative resection for gastric cancer were correlated. We found that prognoses were similar among patients with peritoneal, hematogenous, or nodal recurrences.

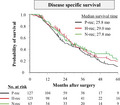

## 1 INTRODUCTION

Patients with advanced gastric cancers often develop recurrences, even if the patients undergo curative resections with D2 lymph node dissection.^1^ Gastric cancer has three predominant recurrence patterns: peritoneal dissemination, hematogenous, and lymph nodal.^2^ Different clinicopathological signatures reportedly include prognosis and treatment response.^3^, ^4^, ^5^, ^6^, ^7^ In the old days, when surgery alone was the standard treatment for advanced gastric cancer, peritoneal recurrence was the most common recurrence pattern in Japan and resulted in the poorest prognosis.^1^

The Adjuvant Chemotherapy Trial of S‐1 for Gastric Cancer (ACTS‐GC) indicated postoperative adjuvant chemotherapy with S‐1 improved prognosis compared with surgery alone for patients with stage II/III gastric cancers.^8^ Decreased peritoneal recurrence was the main cause of the improved prognosis. Few data are available on prognosis after peritoneal, hematogenous, and nodal recurrences after standardization of adjuvant S‐1 treatment. Although several investigators have tried to analyze correlations between initial recurrence sites and prognosis, they have been limited by a small study cohort from a single institution and a long period of data acquisition, accompanied by large changes in disease backgrounds and standard treatment.^3^, ^4^, ^5^, ^6^, ^7^

The objective of our study was to clarify the correlation between initial recurrence site and prognosis after curative resection for gastric cancer using a large‐size multi‐institutional database, acquired between 2010 and 2014.

## 2 PATIENTS AND METHODS

### 2.1 Patient selection

We retrospectively reviewed the medical records of 3484 patients who underwent gastrectomy for gastric cancer between January 2010 and December 2014, at nine participating institutions. The four eligibility criteria were (a) no preoperative treatment; (b) recurrence after R0 gastrectomy with proper systematic lymphadenectomy performed according to the Japanese Gastric Cancer Treatment Guidelines; (c) pathological stage I/II/III gastric cancer according to the 8th edition of TNM staging system of the Union for International Cancer Control; and (d) sufficient data for analysis. Patients who had initial recurrences at multiple sites, local recurrences, or remnant gastric cancers were excluded from the analysis. Patients were classified into three groups based on their site of recurrence at initial presentation; peritoneal, hematogenous, or nodal recurrence.

This study conformed to the ethical guidelines of the World Medical Association Declaration of Helsinki—Ethical Principles for Medical Research Involving Human Subjects. Patients provided written informed consent for surgery and use of clinical data, as required by the Institutional Review Board at each participating institute.

### 2.2 Surgery and postoperative management

The patients underwent gastrectomy with systematic lymphadenectomy according to the Japanese Gastric Cancer Treatment Guideline.^9^ Each patient received postoperative follow‐up that included physical examinations, laboratory tests including serum tumor markers, and enhanced computed tomography (chest and abdominal cavity) once every 6 months for 5 years or until recurrence. S‐1 monotherapy for 12 months or capecitabine plus oxaliplatin for 6 months was recommended for patients with Stage II/III disease (except T1 and T3N0) as postoperative adjuvant treatment unless contraindicated by the patient's condition or by patient refusal. Treatment after recurrence was determined according to evidence available at the time, patient's condition, and patient's consent.

### 2.3 Statistical analysis

Data were analyzed using JMP ver14 software (JMP, SAS Institute). Correlations between each group and clinicopathological variables were analyzed by chi‐square test and Fisher's exact test for categorical variables, and the Mann‐Whitney *U* test for continuous variables. Survival data were calculated using the Kaplan‐Meier method; differences in survival were examined with the Wilcoxon signed‐rank test. *P* < .05 was considered significant.

## 3 RESULTS

### 3.1 Clinicopathologic characteristics and treatment

**FIGURE 1:**
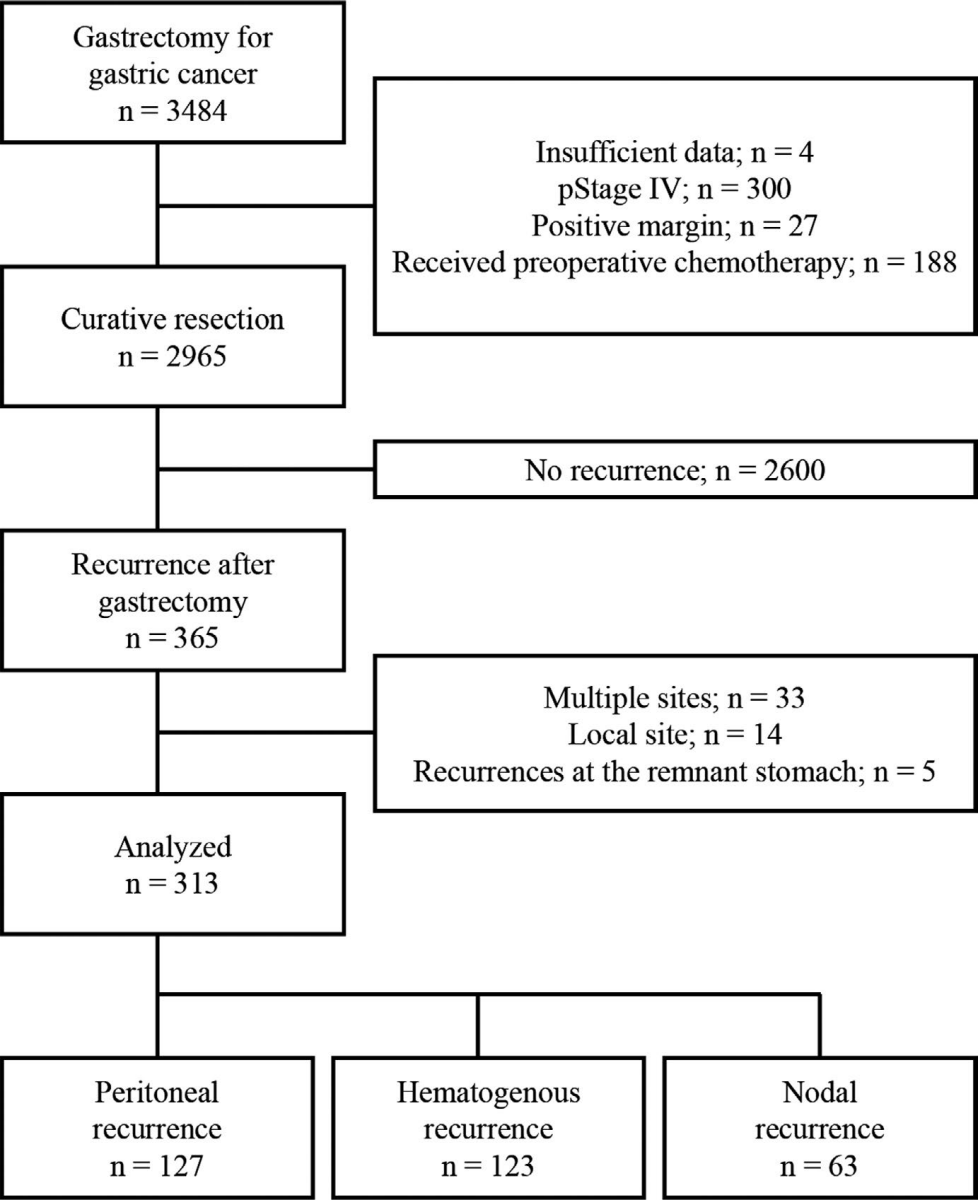
Flow diagram for study participants

A study flow‐chart is presented in Figure 1. Among the 365 patients who developed recurrences, we excluded 52 patients who had multiple site recurrences (n = 33), local recurrences (n = 14), or recurrences at the remnant stomach (n = 5). The remaining 313 patients were classified as having peritoneal (n = 127), hematogenous (n = 123), or nodal recurrences (n = 63). Their characteristics are summarized in Table 1. Patients’ pathological stages were stage I: n = 20 (6%), II: n = 62 (20%), and III: n = 231 (74%). Postoperative adjuvant chemotherapy was administrated to 190 (63%) patients. We compared clinical characteristics between patients who underwent adjuvant chemotherapy and who did not. Patients who did not receive adjuvant chemotherapy had earlier stage, older age, and higher comorbidity rates (Table S1).

**TABLE 1:** Patient characteristics

Variables	n = 313
Age	
Mean ± SD	70.0 ± 9.89
Sex	
Male	220 (70%)
Female	93 (30%)
Method of resection	
Total gastrectomy	153 (49%)
Others	160 (51%)
Tumor location	
Entire	23 (7%)
Upper third	88 (28%)
Middle third	83 (27%)
Lower third	111 (35%)
Remnant	8 (3%)
Macroscopic type	
Borrmann type 4	35 (11%)
Others	278 (89%)
Differentiation	
Differentiated	134 (43%)
Undifferentiated	176 (57%)
pT	
1	24 (8%)
2	22 (7%)
3	94 (30%)
4	173 (55%)
pN	
0	49 (16%)
1	46 (15%)
2	83 (26%)
3	135 (43%)
pStage (UICC 8th)	
Ⅰ	20 (6%)
Ⅱ	62 (20%)
Ⅲ	231 (74%)
Adjuvant chemotherapy	
Absent	111 (37%)
Present	190 (63%)

Abbreviations: SD, standard deviation;UICC, Union for international Cancer Control.

Patients’ clinicopathologic factors by peritoneal, hematogenous, and nodal recurrence groups are presented in Table 2. Patients in the peritoneal group had a significantly greater percentage of younger patients, macroscopic Borrmann type 4, undifferentiated type, invasive growth type, and advanced pT stage. Patients in the hematogenous group had a significantly greater percentage of smaller tumors than the other two groups. The predominant recurrence sites among patients with pT4 tumors were peritoneal (60.1%, 104/173). Clinical characteristics of pStage I patients with postoperative recurrences were summarized in Table S2. Additionally, when focusing on patients with pT1 tumors, the predominant recurrence sites were hematogenous (62.5%, 15/24), followed by nodal (37.5%, 9/24) and no patients with pT1 tumors had peritoneal recurrence.

**TABLE 2:** Comparison of patient characteristics with peritoneal recurrence (P‐rec), hematogenous recurrence (H‐rec), and nodal recurrence (N‐rec)

Variables	P‐rec n = 127	H‐rec n = 123	N‐rec n = 63	*P*‐value		
				P vs H	H vs N	P vs N
Age				<.001	.962	.007
Mean ± SD	67.6 ± 11.0	71.7 ± 8.86	71.6 ± 8.58			
Sex				<.001	.098	.074
Male	74 (58%)	101 (82%)	45 (71%)			
Female	53 (42%)	22 (18%)	18 (29%)			
CEA				.014	.170	.552
≤5	98 (80%)	72 (66%)	42 (76%)			
>5	24 (20%)	37 (34%)	13 (24%)			
CA19‐9				.118	.735	.358
≤37	98 (80%)	78 (72%)	40 (74%)			
>37	24 (20%)	31 (28%)	14 (26%)			
Method of resection				.316	.111	.016
Total gastrectomy	70 (55%)	60 (49%)	23 (37%)			
Others	57 (45%)	63 (51%)	40 (63%)			
Tumor location				.015	.002	.002
Entire	17 (13%)	6 (5%)	0 (0%)			
Upper third	29 (23%)	46 (38%)	13 (20%)			
Middle third	33 (26%)	35 (28%)	15 (24%)			
Lower third	44 (35%)	35 (28%)	32 (51%)			
Remnant	4 (3%)	1 (1%)	3 (5%)			
Tumor size				.005	.016	.887
<50	44 (35%)	64 (52%)	21 (34%)			
≥50	82 (65%)	58 (48%)	41 (66%)			
Macroscopic type				.002	.34	.002
Borrmann type 4	25 (20%)	8 (7%)	2 (3%)			
Others	102 (80%)	115 (93%)	61 (97%)			
Differentiation				<.001	.019	.006
Differentiated	31 (24%)	75 (63%)	28 (44%)			
Undifferentiated	96 (76%)	45 (37%)	35 (56%)			
Lymphatic involvement				.031	.033	.638
Absent	6 (5%)	15 (12%)	2 (3%)			
Present	121 (95%)	108 (88%)	59 (97%)			
Vessel invasion				.885	.881	.788
Absent	31 (24%)	31 (25%)	16 (26%)			
Present	96 (76%)	92 (75%)	45 (74%)			
Infiltrative growth type				<.001	.973	<.001
Invasive growth	87 (70%)	29 (24%)	14 (24%)			
Expansive growth	38 (30%)	90 (76%)	44 (76%)			
pT				<.001	.806	<.001
1	0 (0%)	15 (12%)	9 (14%)			
2	5 (4%)	13 (10%)	4 (7%)			
3	18 (14%)	50 (41%)	26 (41%)			
4	104 (82%)	45 (37%)	24 (38%)			
pN				.002	<.001	.240
0	21 (17%)	23 (19%)	5 (8%)			
1	12 (9%)	28 (22%)	6 (9%)			
2	32 (25%)	38 (31%)	13 (21%)			
3	62 (49%)	34 (28%)	39 (62%)			
pStage (UICC 8th)				<.001	.071	.001
Ⅰ	0 (0%)	14 (11%)	6 (9%)			
Ⅱ	22 (17%)	32 (26%)	8 (13%)			
Ⅲ	105 (83%)	77 (63%)	49 (78%)			
Adjuvant chemotherapy				.002	.223	.192
Absent	34 (28%)	54 (47%)	23 (37%)			
Present	89 (72%)	62 (53%)	39 (63%)			

Abbreviations: CA19‐9, carbohydrate antigen 19‐9; CEA, carcinoembryonic antigen; SD, standard deviation; UICC, Union for international Cancer Control.

### 3.2 Survival outcomes

**FIGURE 2:**
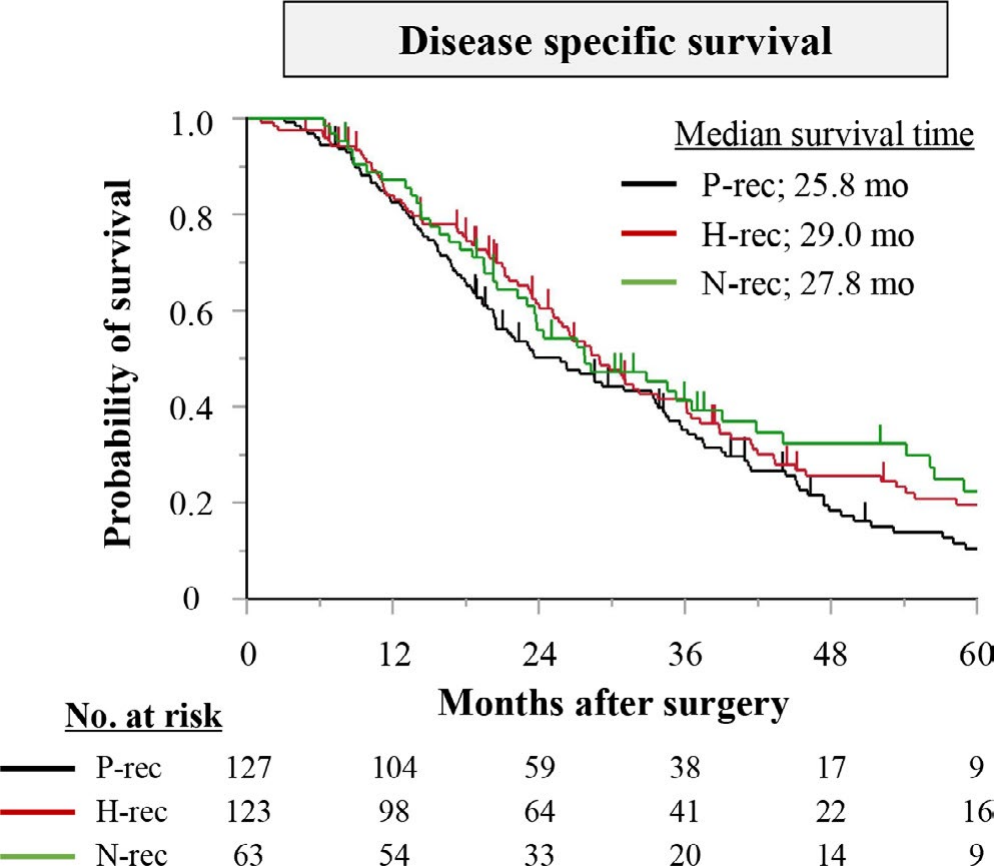
Disease‐specific survival for peritoneal recurrence (P‐rec), hematogenous recurrence (H‐rec), and nodal recurrence (N‐rec) groups. *P < .05

Median follow‐up duration for all 313 patients was 23.8 months (range: 1.2‐114.3 months). Their median disease‐specific survival was 27.8 months (95% CI: 23.8‐31.8). The peritoneal, hematogenous, and nodal recurrence groups did not significantly differ in disease‐specific survival (25.8 vs 29.0 vs 27.8 months; peritoneal vs hematogenous, *P* = .152; hematogenous vs nodal, *P* = .955; peritoneal vs nodal, *P* = .213; Figure 2). Moreover, the subgroup analysis according to adjuvant chemotherapy indicated that disease‐specific survival times after surgery were similar among patients with peritoneal, hematogenous, or nodal recurrences in both subgroups (with and without adjuvant chemotherapy) (Figure S1).

**FIGURE 3:**
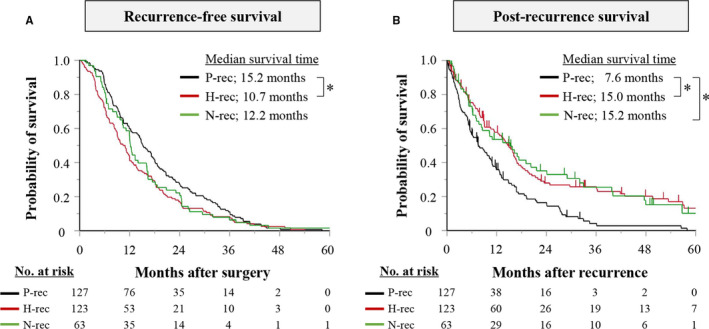
Recurrence‐free survival (A) and post‐recurrence survival (B) for peritoneal recurrence (P‐rec), hematogenous recurrence (H‐rec), and nodal recurrence (N‐rec) groups. *P < .05

Median recurrence‐free survival time was 12.2 months (95% CI: 11.2‐14.1). However, recurrence‐free survival in the peritoneal recurrence group was significantly longer than in the hematogenous recurrence group (15.2 vs 10.7 vs 12.2 months; peritoneal vs hematogenous, *P* = .001; hematogenous vs nodal, *P* = .172; peritoneal vs nodal, *P* = .111; Figure 3A). Median post‐recurrence survival was 10.9 months (95% CI: 9.0‐13.5). Post‐recurrence survival in the hematogenous and nodal recurrence groups did not significantly differ (15.0 vs 15.2 months; *P* = .959), but it was significantly shorter for the peritoneal recurrence group (7.6 months; peritoneal vs hematogenous, *P* < .001; peritoneal vs nodal, *P* = .007; Figure 3B).

## 4 DISCUSSION

In this study, we demonstrated that disease‐specific survival after curative resection of gastric cancer was similar among patients with peritoneal, hematogenous, or nodal recurrences, using a multi‐institutional dataset from patients who underwent gastrectomy after postoperative adjuvant S‐1 became the standard treatment.

Peritoneal metastasis has long been the predominant type of recurrence in the Asia, and it is associated with dismal prognosis because of lack of an effective treatment.^1^ In the last decade, early detection of gastric cancer by health screening systems and standardization of adjuvant treatment have reduced risk of peritoneal recurrence and improved prognosis.^8^, ^10^, ^11^, ^12^ However, percentages of hematogenous and nodal recurrences increased with changes in characteristics of Asian gastric cancer, particularly by eradication of *Helicobacter pylori*, and reduction of peritoneal recurrences by S‐1 adjuvant.^13^, ^14^

In this study, we compared prognosis (disease‐specific, recurrence‐free, and post‐recurrence survival) among patients with peritoneal, hematogenous, or nodal recurrences. Disease‐specific survival times were similar between the three groups, however, recurrence‐free survival in the peritoneal recurrence group was longer than in the hematogenous recurrence group. Additionally, post‐recurrence survival of patients with peritoneal recurrence was shorter than those with hematogenous or lymph nodal recurrence. The longer recurrence‐free survival, but shorter post‐recurrence survival, in the peritoneal recurrence group is interesting. One speculation is that S‐1 adjuvant delays the onset of peritoneal recurrence, but the recurrent disease is more refractory to chemo‐ and radiotherapy and thus associated with shortened survival.^15^ Another is that peritoneal recurrences are diagnosed at later stages with a larger tumor burden because of difficulty in early detection by imaging modalities. Diagnostic methods of disease recurrences had not been prescribed for this study. Because staging laparoscopy is an invasive procedure requiring general anesthesia, it is unrealistic to use it for routine postoperative surveillance. Most of peritoneal recurrences were detected by computed tomography. The sensitivity of computed tomography or fluorodeoxyglucose positron emission tomography for detecting small peritoneal metastasis is low.^16^ Development of more sensitive markers that can enhance the opportunity to detect peritoneal recurrences before appearance of overt ascites fluids or peritoneal nodules could improve this situation.^17^, ^18^, ^19^, ^20^

It is difficult to directly translate our findings into clinical practice. However, data on prognosis after recurrences enable physicians to provide adequate informed consent information to patients having a postoperative recurrence of gastric cancer. Moreover, it is helpful for physicians to estimate postoperative periods that need to be paid special caution for each recurrent pattern and might improve a quality of postoperative surveillance programs.

Some previous reports described relationships between sites of initial recurrence and the clinicopathological characteristics.^3^, ^6^, ^7^ For example, Lee et al reported that patterns of recurrence vary significantly based on Lauren histologic type. They showed that the most common site for intestinal tumors was distant metastasis (54%) and the most common site for diffuse/mixed tumors, was peritoneal recurrence (37%).^6^ Our data indicate that peritoneal recurrence was associated with macroscopic Borrmann type 4, undifferentiated type, invasive growth type, and advanced pT stage. Treatment and surveillance strategies could be tailored to clarify differences in risk factors among recurrence sites.

Takahashi et al reported timing and site‐specific trends of recurrence in a Japanese high‐volume center.^21^ We found that the administration rate of adjuvant chemotherapy was only 64% in those patients probably because of multi‐institutional dataset including elderly patients having comorbidities from regional hospitals. Patients who did not receive adjuvant chemotherapy had earlier stage, older age, and higher comorbidity rates. Moreover, the subgroup analysis according to adjuvant chemotherapy indicated that disease‐specific survival times after surgery were similar among patients with peritoneal, hematogenous, or nodal recurrences in both subgroups (with and without adjuvant chemotherapy). These findings are unique points from a previous study reported by Takahashi et al.^21^

Some limitations associated with this study should be mentioned. First, this is a retrospective analysis. Second, only 63 patients had nodal recurrences. Third, information on treatment after recurrences is insufficient. Fourth, we have no data on disease symptoms or detection methods for the diagnoses of these recurrences.

Taken together, our multi‐institutional dataset analysis revealed that prognosis was similar among patients with peritoneal, hematogenous, and nodal recurrences after curative resection of gastric cancer.

## DATE AVAILABILITY STATEMENT

The datasets during and/or analyzed during the current study are available from the corresponding author on reasonable request.

## CONFLICT OF INTEREST

Nothing to declare.

## AUTHOR CONTRIBUTIONS

KS wrote the manuscript. MK and YK revised the text and contributed to the scientific analysis in the manuscript. SI, YM, HT, KI, TM, TA, AI, HM, CT, DK, and MF contributed to data collection. KM conducted the statistical analyses. All authors have read and approved the final version of the manuscript.

## RESEARCH INVOLVING HUMAN PARTICIPANTS INFORMED CONSENT

The study protocol has been approved by the Institutional Review Board of all participating institutes. The ethical guidelines of the World Medical Association Declaration of Helsinki––Ethical Principles for Medical Research Involving Human Subjects were fully conformed when conducting the present study. A written informed consent for usage of data were granted from all patients before surgery.

## Supporting information

Fig S1

Table S1

Table S2
