# Supplementary figures and images for: Survival times are similar among patients with peritoneal, hematogenous, and nodal recurrences after curative resections for gastric cancer
Source: Cancer Med. 2020 Jun 8;9(15):5392–9. doi: 10.1002/cam4.3208 (PMC7402812; doi:10.1002/cam4.3208)

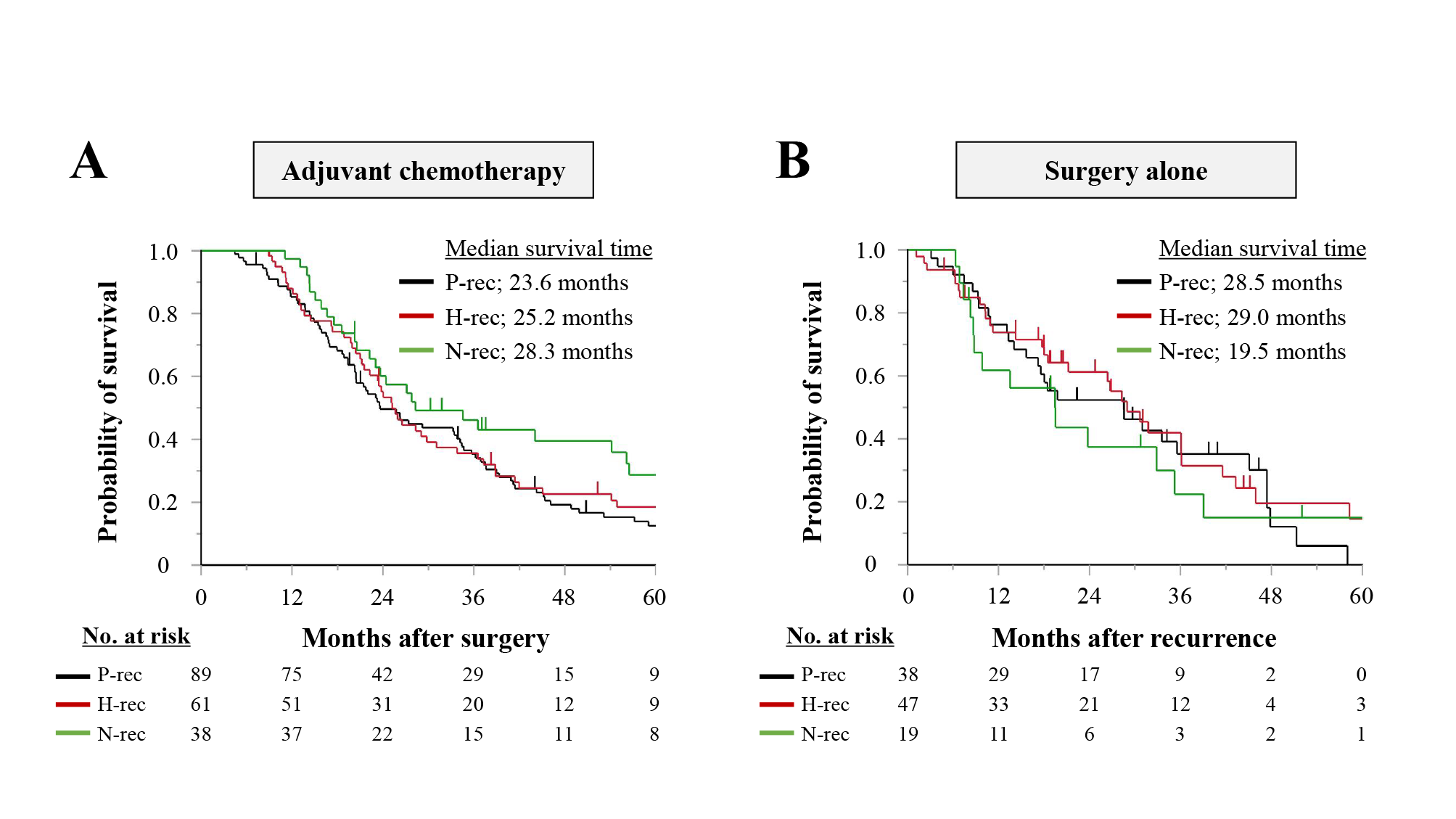

Supplement: Supplementary file 1 — Fig S1 [file CAM4-9-5392-s001.tif]
