# Supplementary material for: Survival times are similar among patients with peritoneal, hematogenous, and nodal recurrences after curative resections for gastric cancer
Source: Cancer Med. 2020 Jun 8;9(15):5392–9. doi: 10.1002/cam4.3208 (PMC7402812; doi:10.1002/cam4.3208)
Supplement: Supplementary file 2 — Table S1 [file CAM4-9-5392-s002.docx]

**Supplemental Table 1.** Comparison of patient characteristics between patients who undergone adjuvant chemotherapy and surgery alone.

| Variables | Adjuvant chemotherapy  n=190 | Surgery alone  n=123 | *P*-value |
| --- | --- | --- | --- |
| Age |  |  | < 0.001 |
| Mean ± SD | 67.8 ± 9.36 | 73.4 ± 9.78 |  |
| Sex |  |  | 0.095 |
| Male | 127 (67%) | 93 (76%) |  |
| Female | 63 (33%) | 30 (24%) |  |
| Performance Status |  |  | < 0.001 |
| 0 | 148 (78%) | 71 (58%) |  |
| 1 | 35 (18%) | 36 (29%) |  |
| 2 | 6 (3%) | 12 (10%) |  |
| 3 | 1 (1%) | 4 (3%) |  |
| Comorbidities |  |  | 0.050 |
| 0 | 77 (41%) | 36 (29%) |  |
| 1 | 55 (29%) | 34 (28%) |  |
| ≥ 2 | 58 (30%) | 53 (43%) |  |
| Method of resection |  |  | 0.977 |
| Total gastrectomy | 93 (49%) | 60 (49%) |  |
| Others | 97 (51%) | 63 (51%) |  |
| Postoperative complications |  |  | 0.579 |
| CD Grade III or higher | 28 (15%) | 21 (17%) |  |
| pStage (UICC 8th) |  |  | < 0.001 |
| I | 2 (1%) | 18 (15%) |  |
| Ⅱ | 32 (17%) | 30 (24%) |  |
| Ⅲ | 156 (82%) | 75 (61%) |  |

*SD* standard deviation, *CD* Clavien-Dindo, *UICC* Union for International Cancer Control.
