# Supplementary material for: Survival times are similar among patients with peritoneal, hematogenous, and nodal recurrences after curative resections for gastric cancer
Source: Cancer Med. 2020 Jun 8;9(15):5392–9. doi: 10.1002/cam4.3208 (PMC7402812; doi:10.1002/cam4.3208)
Supplement: Supplementary file 3 — Table S2 [file CAM4-9-5392-s003.docx]

**Supplemental Table 2.** Characteristics of patients with stage I gastric cancer.

| Variables | Total  n=20 | H-rec  n=14 | N-rec  n=6 |
| --- | --- | --- | --- |
| Age |  |  |  |
| Mean ± SD | 72.9 ± 9.54 | 71.8 ± 10.0 | 75.5 ± 8.54 |
| Sex |  |  |  |
| Male | 17 (85%) | 12 (86%) | 5 (83%) |
| Female | 3 (15%) | 2 (14%) | 1 (17%) |
| Method of resection |  |  |  |
| Total gastrectomy | 3 (15%) | 2 (14%) | 1 (17%) |
| Others | 17 (85%) | 12 (86%) | 5 (83%) |
| Differentiation |  |  |  |
| Differentiated | 15 (75%) | 11 (79%) | 4 (67%) |
| Undifferentiated | 5 (25%) | 3 (21%) | 2 (33%) |
| Lymphatic involvement |  |  |  |
| Absent | 6 (32%) | 4 (29%) | 2 (40%) |
| Present | 13 (68%) | 10 (71%) | 3 (60%) |
| Vessel invasion |  |  |  |
| Absent | 12 (63%) | 8 (57%) | 4 (80%) |
| Present | 7 (37%) | 6 (43%) | 1 (20%) |
| Infiltrative growth type |  |  |  |
| Invasive growth | 2 (13%) | 1 (9%) | 1 (20%) |
| Expansive growth | 14 (87%) | 10 (91%) | 4 (80%) |
| pT |  |  |  |
| 1 | 16 (80%) | 11 (79%) | 5 (83%) |
| 2 | 4 (20%) | 3 (21%) | 1 (17%) |
| pN |  |  |  |
| 0 | 13 (65%) | 10 (71%) | 3 (50%) |
| 1 | 7 (35%) | 4 (29%) | 3 (50%) |
| pStage (UICC 8th) |  |  |  |
| IA | 9 (45%) | 7 (50%) | 2 (33%) |
| IB | 11 (55%) | 7 (50%) | 4 (67%) |

*SD* standard deviation, *UICC* Union for International Cancer Control.
